# Do Physicians CARE? Psychometric Validation of the Portuguese Self-Report Version of the CARE Measure

**DOI:** 10.1177/01632787261422208

**Published:** 2026-01-30

**Authors:** Francisca Rosa, Ricardo Ávila, Ana C. Alves-Nogueira, Marco Pereira, Cláudia Melo, Maria Cristina Canavarro, Carlos Carona

**Affiliations:** 1 Center for Research in Neuropsychology and Cognitive and Behavioral Intervention, Faculty of Psychology and Educational Sciences, 166400 University of Coimbra, Coimbra, Portugal; 2 Cognitive-Behavioral Clinical Psychology Unit (UPC3), University of Coimbra, Coimbra, Portugal

**Keywords:** clinical empathy, physicians, psychometrics, self-report, construct validity, medical education

## Abstract

Clinical empathy is central to the physician–patient relationship, and there is an increasing need for valid and reliable instruments to assess self-reported clinical empathy. Although the Consultation and Relational Empathy (CARE) Measure is widely used in its patient-reported form, it has not been validated for physician self-assessment. This study examined the construct validity and internal consistency of the Portuguese self-report version of the CARE Measure in 221 physicians who completed an online survey. The sample was randomly divided into two subsamples for exploratory and confirmatory factor analyses (EFA and CFA). Measurement invariance was tested across gender, age, and professional status. Internal consistency, convergent validity, and known-groups validity were also evaluated. EFA revealed a two-factor structure – Cognitive and Affective Empathy – supported by CFA. Measurement invariance was confirmed across gender, age, and professional status. The scale showed good internal consistency for both factors. Convergent validity was indicated by correlations with communication competence and therapeutic relationship quality. Known-groups validity was demonstrated by higher cognitive empathy scores among physicians aged 41 and older and among men. These findings provide robust evidence for the validity and reliability of the Portuguese self-report CARE Measure, supporting its use in clinical training, self-assessment, and empathy research.

Effective physician-patient communication is widely recognized as a cornerstone of patient-centered care, playing a critical role in building the therapeutic alliance and directly influencing treatment adherence, patient satisfaction, and clinical outcomes such as symptom improvement, anxiety reduction, and overall therapeutic effectiveness (Alves-Nogueira et al., 2024; Derksen et al., 2013; Roter et al., 2020). Among the core components of physician-patient communication, clinical empathy emerges as a key attribute. The significance of empathy in medical practice is further underscored by its consistent association with positive patient-related outcomes — including enhanced adherence to treatment, greater satisfaction and trust — and with adaptive professional outcomes, such as reduced physician burnout (Hojat, 2016; Howick et al., 2018). Accurate measurement of clinical empathy is therefore valuable for evaluating physician–patient interactions and informing medical education, as it provides a basis for monitoring empathic competencies and guiding initiatives aimed at enhancing communication and care quality.

While most existing instruments prioritize patient-reported assessments of physician empathy, which provide a valuable external perspective, they tend to neglect physicians’ own perceptions of their emotional and cognitive engagement in the clinical encounter (Hojat, 2007). Among these instruments, Consultation and Relational Empathy (CARE) Measure remains one of the most widely used and psychometrically robust tools for assessing empathy in clinical practice, with extensive validation across diverse patient populations and healthcare settings (Mercer et al., 2004). However, to date, no validated self-report version of the CARE Measure has been developed, which limits our understanding of how physicians perceive, regulate, and reflect on their empathic clinical engagement. Importantly, self-reported and patient-reported empathy are related but distinct constructs. Self-report measures provide information about physicians’ own appraisal and regulation of their empathic engagement, whereas patient-reported measures capture how patients experience the interaction. In this context, assessing physicians’ self-reported empathy is valuable not only as a complement to patient-derived information but also as a way to identify possible discrepancies between physicians’ and patients’ perceptions. This distinction supports reflective practice and may inform the development of educational interventions aimed at enhancing empathy in clinical care. To address this gap, we developed and psychometrically evaluated a self-report version of the CARE Measure aimed at providing a reliable tool for assessing physicians’ empathy from their own perspective.

## Clinical Empathy: An Overview of the Concept

Mercer and Reynolds (2002) defined clinical empathy as the capacity to understand the patient’s situation, feelings, and perspective, to communicate that understanding, and to act on it in a helpful manner. This multidimensional conceptualization has gained widespread international acceptance, establishing empathy as a measurable construct in clinical settings. Clinical empathy can be categorized into two core dimensions: cognitive and affective (Hojat, 2016). “Cognitive empathy” refers to the physician’s capacity to understand the patient’s emotional state and perspective, thereby tailoring communication and interventions accordingly (Hojat, 2016; Neumann et al., 2011). “Affective empathy”, on the other hand, entails an emotional resonance with the patient, fostering more compassionate and humane responses (Derksen et al., 2013; Halpern, 2013; Hojat, 2016). Higher levels of physician empathy have been consistently associated with improved therapeutic outcomes, including greater treatment adherence (Zolnierek & DiMatteo, 2009), reduced patient anxiety (Howick et al., 2018), better psychological adjustment (Olaisen et al., 2020), stronger therapeutic alliances and enhanced patient trust (Wu et al., 2021; Zhang et al., 2023). Patients who perceive their physicians as empathetic also report higher satisfaction with care, which may positively influence recovery, overall well-being, and the efficiency of healthcare services (Hojat et al., 2023; Wang et al., 2018; Zhang et al., 2023). In addition to these patient-related outcomes, empathy has also been linked to lower rates of physician burnout, as clinicians who engage empathetically often report greater professional fulfillment and reduced emotional exhaustion (West et al., 2018; Wolfshohl et al., 2019).

However, empathy is not uniformly expressed or understood across all physicians, which may have important implications for medical training and patient outcomes. Research indicates that gender differences can influence both the expression and perception of clinical empathy, with female physicians generally exhibiting higher levels than their male counterparts (Gleichgerrcht & Decety, 2013; Hojat et al., 2023; Neumann et al., 2011). Importantly, patients frequently perceive women as more empathic than men, particularly in affective and relational domains, and these perceptions may not necessarily align with physicians’ self-reported empathy scores (Chen et al., 2020; Roter et al., 2002). These differences may be explained by socialization processes (e.g., gender norms encouraging emotional expressiveness in women; Eagly & Wood, 2012), neurobiological factors (e.g., greater limbic activation in women during empathic tasks; Christov-Moore et al., 2014), and professional role expectations (e.g., relational focus in general practice vs. technical focus in surgery; Halpern, 2007). Moreover, studies indicate that empathy may decline during medical training and across the medical career trajectory, with contributing factors such as increased workload, emotional exhaustion, distressing patient encounters, and the modelling of emotionally detached behaviours by senior physicians (Hojat et al., 2023; Neumann et al., 2011; Stratta et al., 2016). These patterns may intersect with broader contextual and organizational factors that influence wellbeing across the lifespan. For example, burnout tends to peak in the fifth decade of life, potentially reflecting age-related reductions in physiological functioning and increased vulnerability to illness (Batanda, 2024). Organizational stressors, including imbalances in duty allocation, physically strenuous work, and resource constraints, further contribute to burnout and may indirectly shape physicians’ empathic engagement (Imai et al., 2004). Consistent with this, burnout has been consistently associated with lower levels of empathic engagement, underscoring the need to consider both personal and systemic influences on empathy development (Patel et al., 2019; West et al., 2016). In this context, comparing self-report empathy scores across groups defined by age and professional status (i.e., interns/residents vs. specialists) provides valuable insights into how empathic capacities evolve throughout the medical career. This comparison is particularly informative, as it indirectly reflects the combined influence of developmental factors and cumulative clinical experience by contrasting younger, less experienced physicians with older, more experienced ones. Mapping these trajectories is crucial for understanding how this core competency evolves over the course of medical careers and for identifying critical periods in which targeted educational interventions are most needed. Such knowledge can guide the design of training interventions that not only enhance patient care and satisfaction, but also strengthen physician-patient relationships, help prevent professional burnout, and foster greater professional fulfilment among physicians (Hojat et al., 2023; Neumann et al., 2011; Patel et al., 2019).

## The CARE Measure: A Tool for Assessing Clinical Empathy

A variety of proxy-report instruments have been developed to assess clinical empathy, emphasizing the importance of capturing patients’ perspectives on the relational and affective dimensions of care. Among these, the CARE Measure is one of the most widely used. This brief 10-item scale provides an external and relational view of empathic practice and has demonstrated excellent psychometric properties, with high reliability and strong validity evidence (Mercer et al., 2004). The CARE Measure has also been effectively applied to evaluate physicians’ empathic communication and its association with outcomes such as patient satisfaction and treatment adherence (Mercer et al., 2008). Despite these strengths, the CARE Measure is exclusively a patient-reported outcome measure, which limits its ability to capture physicians’ own perceptions and regulation of empathic behavior during consultations.

This limitation underscores the need for self-report instruments that can complement patient perspectives. Existing tools, such as the Jefferson Scale of Empathy (JSE), have been widely employed among healthcare professionals, including both medical students and practicing physicians (Hojat et al., 2001, 2023). However, the JSE and similar measures present notable shortcomings. They rely exclusively on self-assessment, making them vulnerable to social desirability bias, and they focus primarily on the cognitive dimension of empathy, thereby neglecting the affective and interpersonal dynamics that are central to clinical encounters (Neumann et al., 2011). In addition, their relative length can hinder feasibility in time-constrained healthcare contexts.

The development of a self-report version of the CARE Measure addresses these limitations by providing a brief and psychometrically robust tool that allows physicians to appraise their own empathic practice through self-reported empathy scores. When used alongside with the patient-reported version, this approach enables a dual-assessment of empathy, integrating both physician and patient perspectives. Such integration offers a more comprehensive evaluation of clinical empathy, facilitates direct comparisons between self- and patient-perceptions, and informs targeted educational and clinical interventions. Ensuring consistency across the two versions further strengthens the validity of these comparisons and reinforces the utility of the CARE Measure across both research and clinical practice settings (Epstein & Krasner, 2013; Mercer & Reynolds, 2002; Neumann et al., 2011).

## The Current Study

Given the central role of empathy in clinical care and the limitations of existing instruments – such as the cognitive emphasis of the JSE and the patient-centered nature of the original CARE Measure – there is a growing need for tools that capture a broader conceptualization of empathy from the clinician’s own perspective. While the original CARE Measure demonstrated a unidimensional structure through exploratory factor analysis (EFA; Mercer et al., 2004), subsequent work by Gehenne et al. (2020) using confirmatory factor analysis (CFA) supported a three-factor model encompassing cognitive, emotional, and behavioral dimensions, reinforcing the multidimensional nature of empathy in medical practice.

Several translations of the patient-reported CARE Measure have been validated in other linguistic and cultural contexts (e.g., Del Barrio et al., 2020; Neumann et al., 2008), but, to our knowledge, no validated Portuguese patient-reported version currently exists. The present study therefore adapts the CARE framework for physician self-report in Portuguese, maintaining conceptual consistency with the original instrument while capturing physicians’ own perceptions of their empathic engagement through self-reported empathy scores.

In this context, the present study aimed to address the aforementioned research gaps by validating the psychometric properties of a Portuguese self-report version of the CARE Measure in a sample of practicing physicians. Specifically, the internal structure of the scale was first examined through an exploratory factor analysis (EFA), followed by a CFA. To further assess the structural robustness of the measure, measurement invariance across gender (male vs. female), age group (<41 vs. ≥ 41 years), and professional status (interns/residents vs. specialists) was examined using multigroup analyses conducted on an exploratory basis. The reliability of the instrument was assessed through internal consistency estimates, and construct validity was further evaluated via convergent validity (correlations with related constructs) and known-groups validity (comparisons across predefined subgroups).

## Method

### Sample

The study recruited physicians who met the following inclusion criteria: (1) membership in the Portuguese Medical Association; (2) active clinical practice in a Portuguese healthcare institution; (3) status as an intern, resident, or specialist in any medical specialty, and (4) fluency in Portuguese. The exclusion criteria included physicians who were not engaged in clinical practice or who were practicing in other countries.

The sample size calculation was conducted following established guidelines to ensure statistical robustness. Based on recommendations by Hair et al. (2019), a ratio of 5–10 participants per item is suggested for EFA. Given that the self-report version of the CARE scale comprises 10 items, the minimum sample size was estimated to range from 50 to 100 participants. For CFA, which typically requires a larger sample, Kline (2004) recommends a minimum of 100–200 participants to achieve sufficient statistical power. Two subsamples were randomly derived from the total collected sample to meet these criteria and then perform the EFA and the CFA.

### Procedure

The present study is part of a broader project conducted at the host institution, which aimed to develop and assess the feasibility of a novel theory-driven training program to optimize physician-patient communication. Data were collected from September 2022 to April 2023 via an online LimeSurvey® questionnaire (hosted in the website of the host institution) using non-probability convenience and snowball sampling methods. The survey was disseminated via social networks, medical organizations and societies (e.g., Portuguese Medical Association, National Palliative Care Commission, Association of Young Physicians).

The estimated completion time of the survey was 15 min. Informed consent was obtained from all participants, ensuring confidentiality and data protection. To prevent automated responses, a CAPTCHA system was implemented as recommended by Storozuk et al. (2020). Additionally, all questions were mandatory, following recommendations by Smyth et al. (2006), to avoid missing data. Seriousness checks (Aust et al., 2013) were conducted during data preparation for analysis, which included cross-referencing variables such as “age” with “years of service” to identify response inconsistencies. Participants for whom inconsistencies were identified were subsequently excluded from the sample.

The study adhered to the Declaration of Helsinki principles (World Medical Association, 2013) and the Code of Ethics of the Portuguese Psychologists’ Association (Ordem dos Psicólogos Portugueses, 2024)). The project was approved by the Ethics Committee for Research of the Faculty of Psychology and Educational Sciences at the University of Coimbra (CEDI/FPCEUC:64/6).

### Instruments

#### Sociodemographic and Professional Data Questionnaire

A specific self-reported questionnaire was developed by the research team to collect sociodemographic information (e.g., gender, age, educational qualifications) and professional data of the participants (e.g., years of service, medical specialty).

#### Consultation and Relational Empathy Scale–Self-Report (CARE–Self-Report)

The CARE Scale (Mercer et al., 2004) was designed to assess empathy in the physician-patient relationship, focusing on communication and relational behaviors of physicians from the patients’ perspective. To address the lack of a physician self-assessment perspective, this measure was subsequently adapted into a self-report version, enabling the evaluation of physicians’ own perceptions of their empathic behavior during consultations. The Portuguese version of the CARE – Self-Report (Alves-Nogueira et al., 2021a) was developed following internationally recommended procedures for cultural adaptation of health-related measures (Gjersing et al., 2010; Schmidt & Bullinger, 2003). After obtaining authorization from the authors of the original version to translate the questionnaire, the Portuguese version of the CARE – Self-Report was developed through a forward–backward translation procedure. First, the questionnaire was independently translated by two independent researchers, who were fluent in both Portuguese and English and well-acquainted with the concepts and terminology relevant to the questionnaire. Second, those researchers compared the two translated versions and resolved translation disagreements to obtain the first Portuguese version of the CARE – Self-Report. The Portuguese version was subsequently backtranslated into English by a third researcher not familiar with the questionnaire. The original and the back-translated versions were compared to achieve a comprehensible Portuguese version that is conceptually consistent with the original version (Schmidt & Bullinger, 2003). The self-report version measures the physician’s own perception of empathy during consultations. The scale comprises 10 items (e.g., “Do you completely understand the concerns of your patients?”; “Do you show care and compassion?”), rated on a 5-point response scale ranging from 1 (*poor*) to 5 (*excellent*). The total score ranges from 10 to 50 points, with self-reported empathy scores indicating greater self-perceived empathy. Previous studies using the patient-report version demonstrated excellent internal consistency, with Cronbach’s alpha coefficients exceeding .90 (Gehenne et al., 2020; Mercer et al., 2004).

#### Medical Communication Competence Scale (MCCS)

Communication competence was evaluated using the Medical Communication Competence Scale – Doctor Self-Competence (MCCS; Alves-Nogueira et al., 2021b; Cegala et al., 1999). This scale assesses self-perceived medical communication competence across two key dimensions: Information exchange (e.g., “I explained how to use the medication and discussed possible side effects”) and Socioemotional communication (e.g., “I was warm and friendly.”). This scale comprises 24 items rated on a 7-point scale ranging from 1 (*strongly agree*) to 7 (*strongly disagree*). The total score, obtained by summing all items, ranges from 24 to 168, with higher scores reflecting greater levels of physician communication competence. In this study, the scale demonstrated good internal consistency for the total score (α = .91) and for each subscale: information exchange (α = .89) and socioemotional communication (α = .80).

#### Scale to Assess Therapeutic Relationships – Clinician Version (STAR-C)

The STAR-C used in this study (Alves-Nogueira et al., 2022; McGuire-Snieckus et al., 2006) is a 12-item self-report measure that assesses the quality of the therapeutic relationship from the physician’s perspective, based on their interaction with the most recent patient attended. The items are distributed across three subscales: Positive Collaboration (e.g., “I get along well with my patient.”), Emotional Difficulties (e.g., “I find it difficult to empathize with or relate to my patient’s problems.”), and Positive Contribution of the Physician (e.g., “I feel I provide support to my patient.”). The items are rated on a 5-point scale ranging from 0 (*never*) to 4 (*always*), with reversed items on the Emotional Difficulties subscale. A total score, obtained by summing all items, ranges from 0 to 48, with higher scores reflecting better perceived therapeutic relationships. In this study, Cronbach’s alpha was adequate for the total scale (α = .72).

### Data Analysis

The statistical analysis was conducted using SPSS (Version 27.0; IBM SPSS, Chicago, IL) and AMOS (Analysis of Moment Structures; IBM SPSS, Armonk, NY). Descriptive analyses were calculated to characterize the total sample and the subsamples, regarding sociodemographic and professional variables. For analytic purposes, age was dichotomized using the sample median (41 years), ensuring balanced subgroup sizes and adequate statistical power for group comparisons. This value also approximates the 45-year developmental benchmark described by the World Health Organization in the context of health and professional development (Group, 1998), providing an additional conceptual rationale for the chosen cut-off. The resulting age groups were <41 versus ≥41 years.

To assess the suitability of the data for factor analysis, the sampling adequacy was evaluated using the Kaiser-Meyer-Olkin (KMO) measure, which categorizes adequacy as excellent (above .90), very good (.81–.89), good (.71–.80), mediocre (.51–.70), and unacceptable (below .50) (Field, 2013). Bartlett’s Test of Sphericity was conducted to determine whether the correlation matrix is significantly different from an identity matrix, ensuring that the data is appropriate for factor analysis. A significant *p*-value (typically <.05) indicates that the correlation matrix is not an identity matrix, suggesting that the variables are correlated and suitable for factor analysis.

Following this, an EFA was performed to investigate the dimensionality of the scale. Factors were extracted using Principal Component Analysis (PCA), and an Oblimin rotation was applied to account for correlations between factors. The factor retention criteria considered Eigenvalues greater than 1 and scree plot analysis. Additionally, alternative solutions with forced extraction were tested, in alignment with theoretical assumptions. Factor loadings were evaluated, and only those equal to or greater than .30 were considered acceptable, as they indicated meaningful contributions to the factor structure (Field, 2013).

To confirm the factor structure suggested by the EFA, a CFA was subsequently performed. In Structural Equation Modeling (SEM), the chi-square statistic (χ^2^) is a key measure of model fit. A low χ^2^ value, along with a non-significant *p*-value, suggests a good fit between the model and the observed data. Model goodness of fit was further evaluated using standard indices, including χ^2^/df, Comparative Fit Index (CFI), Tucker-Lewis Index (TLI), Normed Fit Index (NFI), and Root Mean Square Error of Approximation (RMSEA). To interpret the fit indices, a CFI, TLI or NFI values higher than .90 was considered indicative of acceptable fit, while values higher than .95 represented an excellent fit. For RMSEA, values of lower than .08 indicated acceptable fit, and values lower than .05 were considered excellent. Similarly, a χ^2^/df ratio lower than two was deemed excellent, and values lower than three were considered acceptable. These cutoffs, along with the criteria for other model fit indices, were based on the guidelines proposed by Hooper (2008), Hu and Bentler (1999) and Kline (2011).

After establishing factorial validity, measurement invariance was explored to determine whether the factor structure was interpreted equivalently across subgroups. Multigroup CFA was conducted across gender (male vs. female), age group (<41 vs. ≥ 41 years), and professional status (interns/residents vs. specialists). Invariance was assessed by comparing constrained and unconstrained models. A non-significant change in chi-square (Δχ^2^) indicated that the constrained model did not significantly worsen model fit, supporting invariance across groups (Little, 2013).

The internal consistency of the scales was assessed using Cronbach’s alpha (α). According to Nunnally and Bernstein (1994), α values equal to or greater than .70 are considered adequate, while values of .80 or above indicate optimal reliability.

Convergent validity was assessed by examining Pearson correlations between the CARE – Self-Report (total and subscales), the MCCS (total and subscales), and the STAR (total). Correlation coefficients were interpreted based on Cohen’s (1988) guidelines: weak (*r* < .30), moderate (.30 ≤ *r* < .50), and strong (*r* ≥ .50).

Finally, known-groups validity was evaluated through comparisons of self-report empathy scores across demographic and professional subgroups (gender, age group, and professional status) using independent-samples *t*-tests. To estimate the magnitude of between-group differences, Cohen’s d was calculated and interpreted as small (*d* = 0.20), medium (*d* = 0.50), or large (*d* = 0.80) (Cohen, 1988).

## Results

### Participants

The obtained global sample comprised 221 participants. Two distinct subsamples were randomly obtained in the SPSS database from the total sample: approximately 30% of the cases (*n* = 80) were randomly assigned, using the select cases procedure, to the EFA subsample, while the remaining participants (around 64%; *n* = 141) were allocated to the CFA subsample. Characterization of both subsamples is described in Supplemental Table 1.

### Exploratory Factor Analysis

A first EFA based on eigenvalues revealed that only the first component had an eigenvalue greater than 1, accounting for 55.2% of the total variance. Examination of the scree plot indicated a steep drop-off after the first component, supporting the notion that a single-factor solution may adequately capture the primary variance in the data. Factor loadings were all above .30, as well as the communalities for all items, further supporting the appropriateness of an unifactorial structure.

A second EFA was conducted based on the theoretical assumption that clinical empathy is organized into two correlated dimensions: cognitive empathy (the capacity to understand the patient’s perspective) and affective empathy (the ability to emotionally resonate with the patient) (Hojat, 2016; Neumann et al., 2011). Two factors were forcibly extracted with an Oblimin rotation, allowing for correlation between the dimensions. Supplemental Table 2 presents the factor loadings and item communalities for the two-factor structure, highlighting the distribution of items across the cognitive and affective dimensions.

The results indicated that Factor 1 explained 55.2% of the variance, while Factor 2 accounted for 9.7%, leading to a cumulative variance of 64.9% explained by the two factors combined. The 10 items demonstrated satisfactory variability and moderate dispersion, as reflected in the descriptive statistics. All factor loadings exceeded .30, and communalities were consistently adequate, confirming the robustness of the factor structure. Factor 1 was labeled “Cognitive Empathy”, as it encompassed items reflecting the physician’s ability to understand and respond to the patient’s concerns in a structured and informative way (e.g., *“*Explain everything clearly?*”*). Factor 2 was labeled “Affective Empathy”, as it captured the emotional quality of the interaction and the ability to build a warm, trusting relationship with the patient (e.g., *“*Show care and compassion?”). The two extracted factors were strongly correlated (*r* = .88, *p* < .01) and with the total scale score (*r* = .95 for Factor 1 and *r* = .88 for Factor 2), supporting the adequacy of a two-factor structure.

An additional EFA was conducted with forced extraction of three factors to explore whether any additional theoretical dimensions not captured in the previous model could emerge. However, the resulting pattern did not yield a clear or theoretically meaningful structure.

### Confirmatory Factor Analysis

A CFA was performed for both unidimensional and bidimensional models of the CARE scale previously identified in the EFA to validate the theoretically derived structures and, ultimately, to attest which model presented a better fit to the data.

The initial unidimensional model presented the following fit indices: χ^2^/*df* = 2.42, CFI = .91, NFI = .86, TLI = .89, RMSEA = .10 90% CI [.07; .13], *p* = .002. The analysis of modification indices suggested possible improvement of fit by correlating the error terms of items 4 and 5, as well as of items 9 and 10, which was found to be reasonable based on the conceptual overlap between those pairs of items. Specifically, the items 4 (“Show interest in your patient as a whole person?”) and 5 (“Fully understand your patients’ concerns?”) relate to a more holistic approach to empathetic patient care, while items 9 (“Help your patient take control?”) and 10 (“Make a plan of action with your patient?”) relate to the development of the patient’s self-determination and internal locus of control in health care. After adding these correlations, the revised model demonstrated improved fit, with χ^2^/df = 1.35, RMSEA = .05 90% CI [.00; .09], *p* = .466, and robust fit indices (CFI = .98, NFI = .93, TLI = .97), indicating an acceptable unidimensional structure.

**Figure 1:**
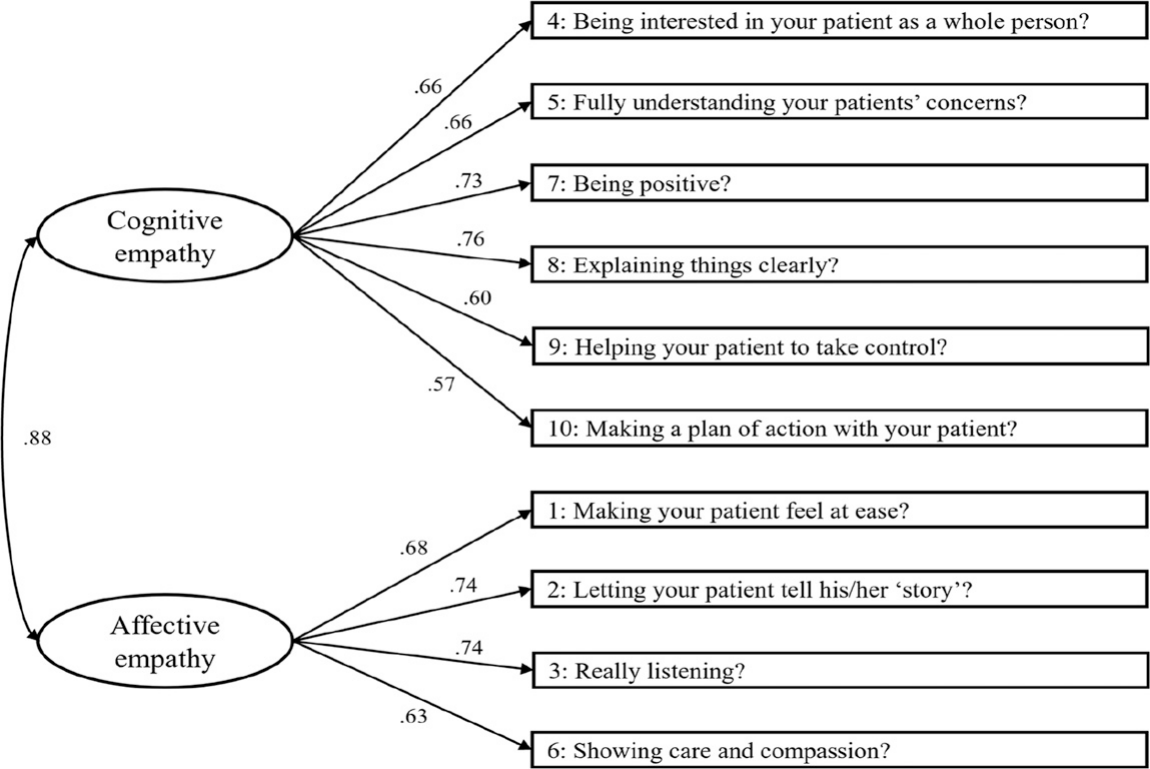
Two-Component Model of the CARE Scale. *Note.* Standardized Regression Weights are Shown; all Were Significant at *p* < .05. Error Terms Were Omitted to Improve Readability

The CFA for the bidimensional model revealed some fit values at the margin of acceptability (χ^2^/*df* = 2.00, CFI = .94, NFI = .89, TLI = .92, RMSEA = .09 90% CI [.06; .11], *p* = .030). Modification indices suggested correlating the same error terms as for the unidimensional model. Therefore, by including those error correlations, the bidimensional model demonstrated an excellent fit to the data, with χ^2^/*df* = 1.10, RMSEA = .03 90% CI [.00; .07], *p* = .770, and excellent fit indices (CFI = .99, NFI = .94, TLI = .99), indicating a robust bidimensional structure. These indices supported the robustness of the bidimensional model. Figure 1 illustrates the two-component structure of the CARE scale, representing the cognitive and affective dimensions of clinical empathy and their observed indicators.

#### Comparison of Unidimensional and Bidimensional Models

To evaluate the parsimony and fit of the models, the Akaike Information Criterion (AIC) and Bayesian Information Criterion (BIC) were calculated. The bidimensional model exhibited lower AIC (81.20) and BIC (149.02) values compared to the unidimensional model (AIC = 88.66, BIC = 153.53), indicating superior parsimony. These results indicate that the bidimensional structure, which separates Cognitive Empathy and Affective Empathy into distinct but correlated factors, provides a more accurate representation of the data compared to the unidimensional structure.

#### Multigroup Analyses

The invariance of the model was tested across gender (male vs. female), age (<41 years vs. ≥ 41 years), and professional status (interns/residents vs. specialists) using an exploratory multigroup analysis. No significant differences were found between the unconstrained models and the nested models, confirming that the model was invariant across all groups. These results are suggestive that the constructs are likely to be interpreted consistently regardless of sex, age, or professional status, as shown in Table 1.

**Table 1:** Multi-Group Analyses to Test Measurement Invariance Across Groups

Group	χ^2^	*df*	CFI	RMSEA [90% CI]	Δχ^2^	Δdf	*p*
Sex							
Male	75.32	32	.73	[.13, .24]			
Female	42.06	32	.98	[.00, .10]			
Unconstrained model	118.08	64	.91	[.06, .10]			
Measurement weights	126.26	72	.91	[.05, .10]	8.18	8	.416
Structural weights	129.57	75	.91	[.05, .09]	11.49	11	.403
Structural covariances	129.57	75	.91	[.05, .09]	11.49	11	.403
Age							
Younger (26–40 years)	37.35	32	.98	[.00, .11]			
Older (41–82 years)	47.34	32	.95	[.02, .12]			
Unconstrained model	84.69	64	.97	[.01, .07]			
Measurement weights	95.22	72	.96	[.01, .07]	10.53	8	.230
Structural weights	97.87	75	.96	[.01, .07]	13.19	11	.281
Structural covariances	97.87	75	.96	[.01, .07]	13.19	11	.281
Professional status							
Interns	46.91	32	.88	[.03, .23]			
Specialists	38.54	32	.99	[.00, .08]			
Unconstrained model	86.57	64	.96	[.02, .08]			
Measurement weights	90.15	72	.97	[.00, .07]	3.57	8	.894
Structural weights	91.19	75	.97	[.00, .07]	4.62	11	.948
Structural covariances	91.19	75	.97	[.00, .07]	4.62	11	.948

### Convergent Validity

The CARE total score demonstrated significant and moderate positive correlations with the MCCS total and subscales, as well as with the STAR total score (cf. Supplemental Table 3). Both the cognitive and affective dimensions showed similar moderate correlations, supporting the expected theoretical associations. These results provide evidence for the convergent validity of the CARE – Self-Report, indicating that higher self-report empathy scores are associated with greater communication competence and higher perceived quality of the therapeutic relationship.

### Known-Groups Validity

Differences in self-reported empathy scores were analyzed across groups of physicians based on gender, age, and professional status, as described in Table 2. The results indicated no significant differences in global empathy between men and women (*p* = .076). However, men scored significantly higher in cognitive empathy compared to women (*t*_(139)_ = 2.31, *p* = .023, Cohen’s *d* = 0.46). No significant differences were observed in affective empathy between genders (*p* = .478). A comparison between younger (<41 years) and older (≥41 years) physicians revealed a marginally significant difference in global empathy, with older physicians scoring higher (*t*_(139)_ = −2.01, *p* = .046, Cohen’s *d* = 0.33). Older physicians also scored significantly higher in cognitive empathy (*t*_(139)_ = −2.61, *p* = .01, Cohen’s *d* = 0.45), while no differences were found in affective empathy between age groups (*p* = .435). Finally, the analysis of professional status (interns/residents vs. specialists) showed no significant differences in empathy scores, whether global, cognitive, or affective.

**Table 2:** Means, Standard Deviations, and Between-Group Comparisons in Clinical Empathy Dimensions Across Sex, Age, and Professional Status

Variables	Sex		Age		Professional status	
	Male	Female	Younger	Older	Interns/residents	Specialists
*n*	39	102	63	78	24	117
Clinical empathy						
*M*	36.7	34.7	34.1	36.2	34.6	35.4
*SD*	5.20	6.47	6.20	6.0	6.20	6.20
*t*	1.80		−2.01*		−0.58	
*d*	0.34		0.33		0.13	
Cognitive empathy						
*M*	22.2	20.5	20.1	21.80	20.1	21.2
*SD*	3.4	4.1	3.9	3.90	3.80	4.0
*t*	2.31*		−2.61**		−1.24	
*d*	0.46		0.45		0.29	
Affective empathy						
*M*	14.5	14.2	14.1	14.4	14.5	14.2
*SD*	2.3	2.9	2.7	2.7	2.7	2.7
*t*	0.67		−0.78		0.50	
*d*	0.13		0.13		0.11	

*Note.* *p < .05; **p < .01.

## Discussion

The self-report version of the CARE Measure revealed a two-factor structure, distinguishing cognitive and affective dimensions of empathy. The measure showed excellent internal consistency, strong construct validity, and measurement invariance across gender, age groups, and professional status. These findings support the CARE – Self-Report as a psychometrically sound tool for assessing physicians’ empathy from their own perspective, with valuable implications for clinical training, professional development, and empathy-related research.

The EFA tested unidimensional, bidimensional, and tridimensional models. Results supported the bidimensional structure – comprising Cognitive and Affective Empathy – as the most appropriate. This model was subsequently confirmed through CFA, which demonstrated excellent model fit. The superiority of the bidimensional model lends support to conceptual models that define clinical empathy as consisting of distinct yet interrelated components (Hojat et al., 2023). While the unidimensional model may capture a general tendency toward empathic behavior, the distinction between “understanding the patient’s perspective” (cognitive empathy) and “experiencing emotional attunement” (affective empathy) provides a more refined account of physicians’ empathetic functioning. The tridimensional model proposed by Gehenne et al. (2020) did not yield a coherent or stable factor solution in this study.

To further assess the robustness and generalizability of this bidimensional structure, measurement invariance analyses were conducted across gender, age, and professional status groups. These analyses confirmed that the bidimensional structure of the CARE – Self-Report was maintained across all subgroups. Factor loadings, intercepts, and residuals were statistically equivalent, indicating that the scale assesses the same constructs in the same way regardless of demographic or professional differences (Hojat et al., 2023; Little, 2013). Although invariance was supported, the fit indices for the male subgroup (CFI = .73) suggested inadequate model fit, likely reflecting the small sample size. This underscores the need for future testing with larger and more representative male samples.

The CARE – Self-Report showed strong internal consistency. Reliability analysis indicated excellent values for the total score and for both dimensions of the scale. Cronbach’s alpha coefficients exceeded conventional cutoffs for adequacy, suggesting strong homogeneity among items and reliable measurement of the underlying constructs (Nunnally & Bernstein, 1994).

Further supporting the validity of the instrument, the CARE – Self-Report also demonstrated good convergent validity, as evidenced by moderate positive correlations with measures of communication competence and the quality of the therapeutic relationship. These associations are theoretically expected, given that empathy is widely recognized as a core component of effective medical communication (Alves-Nogueira et al., 2024; Wang et al., 2018); ) and a key element in fostering strong therapeutic relationships (Wu et al., 2021; Zhang et al., 2023). Specifically, the cognitive dimension of clinical empathy was positively associated with information exchange, a core component of instrumental communication, and with the quality of therapeutic alliances (Wang et al., 2022). In turn, the affective dimension was positively related to socioemotional communication and the overall relational quality of the clinical interaction (Derksen et al., 2018). These associations align with theoretical propositions: cognitive empathy facilitates perspective-taking and accurate information sharing and collaborative decision-making, while affective empathy enhances emotional resonance and interpersonal connection, which are essential to building trust and emotional safety in the clinical encounter (Hojat, 2023; Mercer et al., 2004; Wang et al., 2022).

Regarding known-groups validity, no significant gender differences were observed in global self-reported empathy scores. This contrasts with earlier research reporting higher empathy in women, particularly in affective domains (Christov-Moore et al., 2014; Rueckert et al., 2011), but aligns with more recent studies showing that gender differences are context-dependent and may vary according to specialty or method of assessment (McDonald & Kanske, 2023). Interestingly, male physicians reported higher self-reported cognitive empathy scores than their female counterparts, diverging from traditional expectations. One possible explanation is that gendered norms in professional identity construction shape self-perceptions of empathy, with male physicians expressing confidence in cognitive domains, while female physicians may undervalue or underreport empathic abilities (Kataoka et al., 2009). It is also possible that cognitive empathy, as measured here, aligns more closely with communication behaviors that are socially rewarded in male-dominated medical contexts, thereby amplifying self-reported scores among men (Kataoka et al., 2009).

Similarly, no significant differences were observed between interns/residents and specialists, despite prior research indicating a decline in empathy during medical training (Hojat et al., 2020; Neumann et al., 2011). This discrepancy may be partially explained by the use of a self-report format, as physicians tend to overestimate their empathy levels, particularly in early stages of training, due to limited self-awareness and the influence of social desirability bias (Liang et al., 2022).

In contrast, older physicians reported higher self-reported cognitive empathy scores compared to younger professional peers. This may be attributed to increased clinical experience, improved perspective-taking and the maturation of emotional regulation skills over time (Gleichgerrcht & Decety, 2013; Thomas et al., 2025). Cognitive empathy shows greater plasticity, tipically increasing with age and experience as individuals are exposure to more clinical encounters. Affective empathy tends to remain relatively stable across the lifespan, as it is more closely associated with dispositional emotional traits (Mestre et al., 2009).

The observed findings underscore the practical value of reliably assessing self-reported clinical empathy scores in medical education and professional development. The CARE – Self-Report provides a psychometrically robust instrument that can support formative assessment by enabling physicians to reflect on their relational competencies and identify areas for personal and professional growth. Given its apparent measurement invariance, the scale can be used across diverse demographic subgroups of physicians, regardless of their gender, age, or professional status, to monitor empathy development throughout medical training and clinical practice. Furthermore, the CARE – Self-Report may serve as a formative instrument that can inform the development of empathy-focused educational initiatives, suggesting potential relevance for supporting improvements in physician–patient communication and relational competencies (Derksen et al., 2013).

Despite the contributions and strengths of this study, some methodological limitations should be acknowledged. First, the use of non-probability convenience and snowball sampling, may limit the external validity of the findings. As recruitment was conducted exclusively through online platforms, it is possible that physicians with lower levels of digital literacy or reduced engagement with digital tools may not have accessed the study invitation, thereby reducing the representativeness of the sample (Topolovec-Vranic & Natarajan, 2016). Moreover, as the sample was drawn from a Western European cultural context, the generalizability of the results to other sociocultural settings may be constrained. Second, the exclusive reliance on self-report measures increases the likelihood of social desirability bias and may lead to overestimations of physicians’ competencies. These methods depend on individuals’ self-awareness and introspective accuracy, which may be limited-particularly in domains such as emotional responsiveness-thus potentially failing to reflect what participants actually feel, think, or do (Latkin et al., 2017). This limitation is further compounded by the absence of complementary proxy data sources, such as patient-reported outcomes or observer ratings. Moreover, because the study relied exclusively on self-reported empathy scores, it is not possible to determine whether physicians’ self-perceptions correspond to patients’ evaluations of physician’s clinical empathy. This limitation underscores the need for future research to incorporate patient-reported CARE data, which would enable a more comprehensive examination of the concordance between physicians’ perceived empathy and the corresponding patients’ appraisals. Third, although age-based comparisons were conducted, age and years of service were highly correlated in this sample, with a correlation coefficient of approximately .95, which is near to a perfect positive correlation. This indicates substantial overlap between developmental and professional trajectories. Importantly, chronological age does not necessarily correspond to professional experience, training background, or exposure to communication skills. Age has been described as a primary developmental marker (Holmbeck, 2002), and empathy develops across the lifespan in response to multiple contextual influences (Dorris et al., 2022). By contrast, years of practice reflect exposure to communication training, specialty-specific socialisation, and the cumulative acquisition of clinical experience. For this reason, age was used in the present study as a pragmatic proxy for clinical experience rather than as a strict developmental indicator. Because the study did not analytically disentangle developmental and professional influences, age-related findings should be interpreted with particular caution. This prudence is further warranted by the mixed findings reported in previous research. Some studies reported higher cognitive empathy among older physicians (e.g., Thomas et al., 2025), whereas others found stronger empathy scores among younger physicians, particularly those early in training (e.g., Hojat et al., 2009). The present findings therefore align with some prior results while diverging from others, underscoring the need for future studies to examine age and years of practice as independent variables. Fourth, the cross-sectional design of the study precludes the examination of temporal stability or responsiveness to interventions (e.g., empathy training), which are critical considerations if the instrument is to be employed in longitudinal research or evaluative contexts (Roth & Altmann, 2020). Finally, although measurement invariance was demonstrated across genders, age groups, and professional status, these multigroup analyses were exploratory and should be interpreted with caution. The modest sample size and unequal sizes of the subgroups may have affected the stability and sensitivity of the model comparisons, potentially limiting the generalization of the invariance results.

Future research should adopt longitudinal designs to evaluate the temporal stability and sensitivity to change of the CARE – Self-Report. These designs would allow testing whether clinical empathy, as self-perceived by physicians, remains stable over time or responds to interventions, such as empathy training programs. Importantly, future studies could integrate self-reports data with external sources – such as patient evaluations – to assess the extent to which perceived improvements in empathy correspond to enhancements in patient-perceived relational quality. Because validating a self-report measure represents only an initial step, future research should examine the extent to which physicians’ self-reported empathy corresponds to patients’ evaluations of empathic communication. Integrating patient-reported CARE data will be essential to determine whether self-perceived empathy meaningfully reflects empathic behaviour in clinical practice. In addition, further observational research will be necessary to assess how physicians’ empathic behaviors manifest during real clinical encounters, since self-report alone cannot capture actual relational performance (e.g., Noordman et al., 2019). Future studies should also evaluate additional psychometric properties of the CARE Self-Report, including temporal stability, sensitivity to change in pre–post training designs, and the performance of equivalent forms. These steps are crucial for determining whether the instrument can reliably detect changes over time and whether improvements in self-reported empathy correspond to measurable changes in patient experience and clinical interaction quality. In addition, longitudinal studies could explore the predictive value of the scale by examining whether baseline levels of cognitive and affective empathy predict key clinical or professional outcomes, including patient satisfaction, adherence to treatment, or physician well-being. Further research should also explore potential mechanisms of change for instance, whether empathy mediates the effects of communication training on patient outcomes and examine moderating variables, such as physician gender or years of clinical experience, to better understand for whom and under which conditions such interventions are most effective. Altogether, the present study provides foundational psychometric evidence for the CARE – Self-Report, supporting its reliability, validity, and bidimensional structure in assessing physicians’ self-reported empathy. By offering a brief and theoretically informed instrument, this work strengthens the assessment of empathy skills in medical practice and establishes a basis for future research. Further studies are needed to examine how self-reported empathy aligns with patient-reported outcomes and to explore its role within broader developmental and professional trajectories.

## Supplemental Material

Supplemental Material - Do Physicians CARE? Psychometric Validation of the Portuguese Self-Report Version of the CARE Measure Supplemental Material for Do Physicians CARE? Psychometric Validation of the Portuguese Self-Report Version of the CARE Measure by Francisca Rosa, Ricardo Ávila, Ana C. Alves Nogueira, Marco Pereira, Cláudia Melo, Maria Cristina Canavarro, and Carlos Carona in Evaluation & the Health Professions

## Data Availability

The data that support the findings of this study are available from the senior author (ccarona@fpce.uc.pt) upon reasonable request.
