# Supplementary material for: Do Physicians CARE? Psychometric Validation of the Portuguese Self-Report Version of the CARE Measure
Source: Eval Health Prof. 2026 Jan 30;49(3):285–98. doi: 10.1177/01632787261422208 (PMC13379610; doi:10.1177/01632787261422208)
Supplement: Supplemental Material - Do Physicians CARE? Psychometric Validation of the Portuguese Self-Report Version of the CARE Measure [file sj-pdf-1-ehp-10.1177_01632787261422208.pdf]

Supplementary Table 1

Sociodemographic and Professional Characteristics of EFA (n = 80) and CFA Subsamples (n= 141)

	EFA subsample	CFA subsample
**Sex** (*n*/ %)		
Male	29 (36.3)	39 (27.7)
Female	51 (63.7)	102 (72.3)
**Age** (*M* (*SD*); range)	45.6 (15.7); 25-82	46.3 (14.4); 26-82
**Professional status** (*n*/%)		
Interns/Residents	16.0 (20.0)	24.0 (17.0)
Specialists	64.0 (80.0)	117.0 (83.0)
**Medical specialty** (*n*/%)[a]		
Anesthesiology	1 (1.6)	4 (3.4)
Child/Adolescent Psychiatry	1 (1.6)	2 (1.7)
Dermatology	-	2 (1.7)
Endocrinology and Nutrition	-	1 (0.9)
Forensic Medicine	-	1 (0.9)
Gastroenterology	-	1 (0.9)
General and Family Medicine	22 (34.4)	31 (26.5)
Gynecology/Obstetrics	8 (12.5)	9 (7.7)
Immunohematology	-	1 (0.9)
Infectiology	-	2 (1.7)
Internal Medicine	5 (7.8)	15 (12.8)
Medical Oncology	4 (6.3)	4 (3.4)
Nephrology	1 (1.6)	2 (1.7)
Occupational Medicine	-	1 (0.9)

Ophthalmology	-	1 (0.9)
Orthopedics	3 (4.7)	3 (2.6)
Otorhinolaryngology	-	1 (0.9)
Pediatrics	5 (7.8)	7 (6.0)
Psychiatry	4 (6.3)	10 (8.5)
Public Health	1 (1.6)	1 (0.9)
Pulmonology	3 (4.7)	3 (2.6)
Rheumatology	-	3 (2.6)
Stomatology	1 (1.6)	2 (1.7)
Surgery[b]	4 (6.3)	7 (6.0)
**Years of Service** (*M* (*SD*); range)	18.4 (13.9); 1-43	19.11 (13.4); 0-50
**Healthcare institution** (*n*/%)		
Public	67 (83.8)	115 (81.6)
Private	8 (10.1)	19 (13.5)
Other	5 (6.3)	7 (5.0)

Note. [a] When referencing medical specialties, only physicians holding a specialist degree were considered. [b] Includes all surgical specialties (e.g., general surgery, maxillofacial surgery).

Supplementary Table 2

Factor Loadings and Communalities for Two-Dimension CARE

Item	M (SD)	Cognitive empathy	Affective empathy	Communalities (h^2)
10: Making a plan of action with your patient?	3.40 (0.98)	**.898**	-.195	.65
5: Fully understanding your patients' concerns?	3.49 (0.89)	**.804**	.075	.74
7: Being positive?	3.55 (0.84)	**.757**	.057	.69
4: Being interested in your patient as a whole person?	3.53 (1.09)	**.733**	.143	.68
9: Helping your patient to take control?	3.23 (0.95)	**.564**	.301	.72
8: Explaining things clearly?	3.60 (0.81)	**.554**	.341	.48
1: Making your patient feel at ease?	3.71 (0.81)	-.100	**.859**	.63
3: Really listening?	3.38 (0.86)	.051	**.800**	.65
2: Letting your patient tell his/her 'story'?	3.39 (0.86)	.148	**.760**	.61
6: Showing care and compassion?	3.43 (0.98)	.077	**.647**	.64

Supplementary Table 3

Correlations Between CARE Measure (Total and Dimensions) and Other Measures

CARE	MCCS Total	MCCS Socioemotional	MCCS Information Exchange	STAR Total
Total	.52	.48	.47	.52
Cognitive	.54	.44	.50	.50
Affective	.39	.43	.32	.45

Note. *p* < .01.
